# Novel secondary metabolite from a new species of *Hypoxylon saxatilis* sp. nov. for suppressing bacterial wilt in tomato

**DOI:** 10.1016/j.crmicr.2025.100445

**Published:** 2025-07-20

**Authors:** Thanapat Suebrasri, Wasan Seemakram, Awat Wisetsai, Thanawan Gateta, Sutarin Preepram, Phornnapa Saentao, Sophon Boonlue

**Affiliations:** a Division of Basic and Preclinical Science, Institute of Science and General Education, Nakhon Ratchasima College, Nakhon Ratchasima 30000, Thailand; b Department of Microbiology and Parasitology, Faculty of Medical Science, Naresuan University 65000, Thailand; c Department of Microbiology, Faculty of Science, Khon Kaen University, Khon Kaen 40002, Thailand; d Department of Industrial Chemistry, Faculty of Applied Science, King Mongkut’s University of Technology North Bangkok, Bangkok 10800, Thailand; e Department of Chemistry and Center for Innovation in Chemistry, Faculty of Science, Khon Kaen University, Khon Kaen, Thailand

**Keywords:** Bactericidal metabolites, Disease suppression, New secondary metabolite, Plant pathogen, Tetrahydrofuran

## Abstract

The bacterium *Ralstonia solanacearum* is an important pathogen that causes wilt disease in tomatoes, leading to a reduction in tomato yield. Therefore, this study aimed to investigate the antibacterial activity of endophytic fungi isolated from medicinal plants against the wilt-causing bacterium *R. solanacearum* in tomatoes under both laboratory and greenhouse conditions. Forty-eight fungi were isolated from 20 medicinal plant species using the surface sterilization technique. The antagonistic activity against *R. solanacearum* was subsequently examined using the agar plug diffusion test. Two isolates, *Hypoxylon* sp. NGPM and *Hypoxylon saxatilis* KKU-KHP 01, were identified from the midrib of the *Acanthus ebracteatus* and the petiole of the *Gardenia saxatilis* (Geddes), respectively. Furthermore, the biologically active compounds extracted from both fungal endophytes inhibited the pathogen in the fungal fermentation broth. Notably, *H. saxatilis* KKU-KHP 01 is a newly identified fungal species and the first within its genus to produce tetrahydrofuran, a novel compound not previously reported as a natural secondary metabolite. This compound exhibits inhibitory activity against the phytopathogen *R. solanacearum*. Scanning electron microscopy revealed that exposure to pure tetrahydrofuran caused structural damage to the bacterial cell wall, resulting in visibly wrinkled cells. Additionally, under greenhouse conditions, the crude extract of *H. saxatilis* KKU-KHP 01 could reduce the severity of wilt disease by up to 83.33%, effectively caused by tetrahydrofuran. Therefore, tetrahydrofuran can be applied to control wilt disease in tomatoes under field conditions and can be further developed as a commercial biocontrol agent.

## Highlights

• We reported a new endophytic species with bactericidal activity against Ralstonia solanacearum.
• We elucidated that tetrahydrofuran is produced by the newly identified endophytic fungus Hypoxylon saxatilis.
• We were the first to identify tetrahydrofuran as a previously undescribed secondary metabolite from natural sources.
• We were the first to report tetrahydrofuran bactericidal compounds effective against R. solanacearum.

## Graphical abstract

**Figure:**
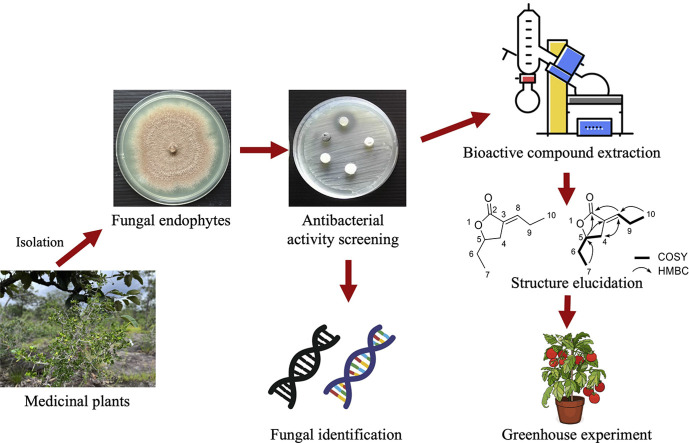

## 1 Introduction

Tomato (*Lycopersicon esculentum* Mill.) is a fruit widely consumed worldwide. It is popular for use in various types of dishes, such as tomato sauce, salads, and soups, and as ingredients in many other foods, both fresh and processed (Liu et al., 2024). It is often produced on an industrial scale to make products that are used in daily life. In 2024, global consumption of tomatoes, including both fresh and processed forms, was estimated to have reached approximately 45.7 million tons, with the majority coming from processed products such as tomato sauce and tomato juice (World Processing Tomato Council, 2024). the main areas for the cultivation of tomatoes in Thailand are in the northern and northeastern regions. According to annual tomato cultivation statistics from the Northeast Region of the Department of Agricultural Extension, the area for tomato production increased from 2490.24 hectares in 2019 to 6328.8 hectares in 2020 (Office of Agricultural Economics, 2020). However, tomatoes are invaded by pests and diseases caused by microorganisms, which can lead to reduced yields, especially bacterial wilt diseases caused by Ralstonia solanacearum. It is one of the most significant plant pathogens worldwide, infecting over 450 host species. It colonizes the xylem vessels of various plants, leading to bacterial wilt disease. Its host range encompasses numerous plant species, including tobacco, bananas, peppers, potatoes, geraniums, and especially tomatoes (Vailleau and Genin., 2023). Bacterial wilt is prevalent in tropical, subtropical, and temperate regions, causing substantial economic losses due to yield reduction and the costs associated with disease management (Wicker et al., 2007).

Several chemicals, such as copper-based bactericides, copper hydroxide, copper oxychloride (Boucher et al., 2005), and sodium hypochlorite (Leelaporn et al., 2008), have been used to control bacterial wilt caused by *R. solanacearum*. Chemicals can be used to eliminate bacterial wilt disease. The application of these chemicals must be approached with caution, as improper use or excessive quantities can lead to adverse effects on both the environment and the health of consumers and farmers. Currently, biocontrol is becoming increasingly popular because of its safety for consumers and its environmentally friendly nature. Endophytic microbes are another promising alternative for controlling plant pathogens, as they reside within plant tissues without harming the host (Chauhan et al., 2024). Furthermore, they can promote plant growth and produce secondary metabolites that inhibit the growth of pathogens (Kloepper et al., 2018; Singh et al., 2020). In addition, endophytic fungi isolated from medicinal plants are selected over those from diseased host plants due to the well-documented diversity of endophytes in medicinal plants capable of producing bioactive secondary metabolites. These endophytic fungi often possess antimicrobial properties that enhance the natural defense mechanisms of their host plants (Strobel and Daisy, 2003). Therefore, medicinal plants represent a promising source for isolating endophytic fungi with potential applications in the development of novel biocontrol agents for plant disease management. Previous reports have indicated that the endophytic bacteria *Bacillus subtilis* (VSB-1) and *Streptomyces thermodiastaticus* (ORA-1) are effective against bacterial wilt under pot culture conditions (Agarwal et al., 2021). The endophytic root microbiomes, including *Pseudomonas simiae* E18, *Bacillus amyloliquefaciens* E60, and *Paenibacillus polymyxa* E29, are also highly effective against *R. solanacearum* (Zhang et al., 2022). In addition, important natural bactericidal compounds, such as 2-azaanthraquinone derivatives produced by the endophytic fungus *Lophiostoma* sp. (Mao et al., 2021) and lipopeptide secreted from *Bacillus subtilis* R31, effectively inhibit the growth of *R. solanacearum* (Li et al., 2023). However, the role of endophytic fungi and their secondary metabolites in controlling bacterial wilt in tomatoes caused by *R. solanacearum* is poorly understood. Moreover, the secondary metabolites that exhibit efficacy in reducing bacterial wilt incidence hold promising potential for future commercial application. Therefore, the present work aimed to isolate and identify endophytic fungi from medicinal plants and determine the ability of endophytic fungi to produce bioactive compounds for controlling *R. solanacearum*. Furthermore, the bioactive compounds isolated from endophytic fungi were subjected to structural elucidation. Their potential to protect tomato plants from *R. solanacearum* infection was evaluated under greenhouse conditions. The findings suggest that these compounds hold promise as biological control agents against bacterial wilt, offering a sustainable approach to enhance tomato yield in the future.

## 2 Materials and methods

### 2.1 Collection of plant materials

A total of twenty medicinal plants were collected from the mountain in Lao Hai Ngam Subdistrict, Kuchinarai District, Kalasin Province, Thailand (16°45′06.3″N 104°22′00.2″E). The plant samples were stored in a sterilized plastic bag at 4 °C until they were processed.

### 2.2 Isolation of endophytic fungi

The plant samples were cleaned with tap water to remove dust. Then, they were cut into pieces with a size of 1–2 mm. The plant segments were sterilized by first soaking them in 70 % (v/v) ethanol for 3 min, followed by immersion in 5 % (v/v) sodium hypochlorite for 1 min. Afterward, the segments were rewashed with 70 % (v/v) ethanol for 3 min, rinsed three times with sterile distilled water, and dried on sterile paper under airflow to ensure complete drying. The sterilized samples were then placed onto half-strength potato dextrose agar (PDA) plates supplemented with 2000 mg/L chlortetracycline to inhibit the growth of endophytic bacteria. Fungi growing from the segment samples were subcultured onto fresh PDA media until a pure culture was obtained. Finally, to confirm the absence of contamination on the sterilized plant segments, an aliquot of the sterilizing solution was placed on agar plates and incubated for 2 weeks to ensure that no fungal growth occurred (Suebrasri et al., 2020).

### 2.3 Screening endophytic fungi as biological control agents against *R. solanacearum*

The endophytic fungi were screened for their ability to antagonize the pathogenic bacteria *R. solanacearum* using the agar plug diffusion method. The bacteria were cultivated in nutrient broth and shaken at 180 rpm at 37 °C for 24 h. After incubation, the turbidity of the bacterial culture was adjusted to 0.5 McFarland, and the bacteria were spread on nutrient agar. Five-day-old fungal endophytes grown on potato dextrose agar media were plugged and placed on the surface of nutrient agar plates, which were spread with the bacteria *R. solanacearum*. A clear zone around the colony of fungal endophytes was observed after incubation at 37 °C for 5 d

### 2.4 Fungal endophyte identification

Fungal endophyte isolates capable of inhibiting the bacterial pathogen *R. solanacearum* were initially identified based on their morphology using light microscopy. Additionally, the fungal colony morphology was observed by culturing on an oatmeal agar plate. To confirm fungal identification, gene sequencing targeting universal primers, including the internal transcribed spacer regions (ITS1–4) and β-tubulin 2 (TUB2), was performed. The primer sequences for the ITS gene and β-tubulin 2 gene were as follows: ITS1 (5′-TCC GTA GGT GAA CCT GCG G-3′), ITS4 (5′-TCC TCC GCT TAT TGA TAT GC-3′), Btub2 (5′-GTB CAC CTY CAR ACC GGY CAR TG-3′), and Btub4 (5′-CCR GAY TGR CCR AAR ACR AAG TTG TC-3′). PCRs were carried out in a total volume of 25 µL, consisting of 5 µL of 10 × PCR buffer, 1.5 µL of 25 mM MgCl₂, 0.5 µL of 1.25 mM dNTPs, 1.5 µL of each primer (150 µM), 100 ng of fungal DNA template, and 0.2 µL of Taq DNA polymerase (5 U/µL) (Thermo Fisher Scientific, Lithuania, EU). The volume was adjusted to 25 µL with sterile distilled water. PCR amplification was performed using a thermal cycler under the following conditions: initial denaturation at 95 °C for 5 min; 35 cycles of denaturation at 95 °C for 1 min, annealing at 52 °C for 1 min, and extension at 72 °C for 90 s; and a final extension at 72 °C for 10 min. The PCR products were purified using the GeneJET™ PCR Purification Kit (Fermentas, Canada). The PCR products were subsequently sequenced with the 1st BASE DNA Kit (Malaysia). Subsequently, the nucleotide sequences were analyzed using the BLAST tool available on the NCBI website.

The authentic DNA sequences from GenBank (http://www.ncbi.nlm.nih.gov) and those obtained from the PCR products were combined with the novel sequences generated in this study, as shown in Table 1. *Xylaria hypoxylon* CBS 122,620 and *X. arbuscula* CBS 126,415 were used as the outgroup. Multiple sequence alignment was performed using MUSCLE (Edgar, 2004) with default settings, and adjustments were made as needed using BioEdit version 6.0.7 (Hall, 2013). The combined alignment of the ITS and *β-Tubulin* sequences was deposited in TreeBASE.

**Table 1:** Details of the sequences used in the molecular phylogenetic analyses in this study. The samples and sequences obtained in this study are in bold.

Hypoxylon species	Strain/Isolate	Country	GenBank accession number		References
			ITS	*β-Tubulin*	
*Annulohypoxylon annulatum*	CBS 140,775	USA	KU604559	KX376353	Kuhnert et al. (2017)
*A. moriforme*	CBS 123,579	Martinique	KX376321	KX271261	Kuhnert et al. (2017)
*Daldinia dennisii*	CBS 114,741	Australia	JX658477	KC977262	Stadler et al. (2014)
*Hypoxylon anthochroum*	YMJ 9	Mexico	JN660819	AY951703	Hsieh et al. (2005)
*H. aveirense*	CMG 29 T	Portugal	MN053021	MN066636	Vicente et al. (2021)
*H. baruense*	UCH9545 T	Panama	MN056428	MK908142	Cedeño-Sanchez et al. (2020)
*H. begae*	YMJ 215	USA	JN660820	AY951704	Hsieh et al. (2005)
*H. brevisporum*	YMJ 36	Puerto Rico	JN660821	AY951705	Hsieh et al. (2005)
*H. carneum*	MUCL 54,177	France	KY610400	KX271270	Wendt et al. (2018)
*H. chrysalidosporum*	FCATAS2710 T	China	OL467294	OL584229	Ma et al. (2022)
*H. cyclobalanopsidis*	FCATAS2714 T	China	OL467298	OL584232	Ma et al. (2022)
*H. diperithecium*	FCATAS 4226 T	China	ON178671	ON365565	Zhu et al. (2024)
*H. diperithecium*	FCATAS 4323	China	ON178672	ON365566	Zhu et al. (2024)
*H. erythrostroma*	YMJ 90,080,602	China	JN979416	AY951716	Hsieh et al. (2005)
*H. ferrugineum*	CBS6 141,259	Austria	KX090079	KX090080	Friebes G & Wendelin I (2016)
*H. fraxinophilum*	MUCL 54,176	France	KC968938	KC977301	Kuhnert et al. (2014)
*H. fulvosulphureum*	MFLUCC 13–0589 T	Thailand	KP401576	KP401584	Sir et al. (2015)
*H. griseobrunneum*	CBS 331.73 T	India	KY610402	KC977303	Kuhnert et al. (2014)
*H. hematostroma*	MUCL 53,301 ET	Martinique	KC968911	KC977291	Wendt et al. (2018)
*H. hainanense*	FCATAS2712 T	China	OL467296	OL584231	Ma et al. (2022)
*H. hinnuleum*	MUCL 3621 T	MUCL 3621	MK287537	MK287575	Daranagama et al. (2015)
*H. hypomiltum*	MUCL 51,845	Guadeloupe	KY610403	KX271249	Wendt et al. (2018)
*H. invadens*	MUCL 51,475 T	France	MT809133	MT813038	Becker et al. (2020)
*H. investiens*	CBS 118,183	Malaysia	KC968925	KC977270	Wendt et al. (2018)
*H. lateripigmentum*	MUCL 53,304 T	Martinique	KC968933	KC977290	Wendt et al. (2018)
*H. lividicolor*	YMJ 70	China	JN979432	AY951734	Helaly et al. (2018)
*H. lividipigmentum*	YMJ 233	Mexico	JN979433	AY951735	Hsieh et al. (2005)
*H. macrocarpum*	CBS119012	Germany	ON792785	ON813071	Helaly et al. (2018)
*H. musceum*	MUCL 53,765	Guadeloupe	KC968926	KC977280	Kuhnert et al. (2014)
*H. notatum*	YMJ 250	USA	JQ009305	AY951739	Hsieh et al. (2005)
*H. papillatum*	ATCC 58729T	USA	NR155153	KC977258	Wendt et al. (2018)
*H. perforatum*	CBS 115,281	France	KY610391	KX271250	Wendt et al. (2018)
*H. pilgerianum*	STMA 13,455	Martinique	KY610412	KY624315	Wendt et al. (2018)
*H. pseudofendleri*	MFLUCC 11–0639	Thailand	KU940156	N/A	(Dai et al. (2017)
*H. pseudofuscum*	18,264 T	Germany	MW367857	MW373867	Lambert et al. (2021)
*H. pulicicidum*	CBS 122622T	Martinique	JX183075	JX183072	Bills et al. (2012)
*H. rickii*	MUCL 53,309 ET	Martinique	KC968932	KC977288	Wendt et al. (2018)
***H. saxatilis***	**KKU-KHP01**	**Thailand**	**LC872603**	**LC872604**	**This study**
*H. sporistriatatunicum*	UCH9542T	Panama	MN056426	MK908140	Cedeño-Sanchez et al. (2020)
*H. sublenormandii*	JF 13,026	Sri Lanka	KM610291	KM610303	Kuhnert et al. (2015)
*H. teeravasati*	PUFD 4T	India	N/A	MG986894	Phookamsak et al. (2019)
*H. texense*	DSM 107933T	USA	MK287536	MK287574	Sir et al. (2019)
*H. trugodes*	MUCL 54794T	Sri Lanka	KF234422	KF300548	Kuhnert et al. (2014)
*H. ulmophilum*	YMJ 350	Russia	JQ009320	AY951760	Hsieh et al. (2005)
*H. vogesiacum*	CBS 115,273	France	KC968920	KX271275	Wendt et al. (2018)
*H.wuzhishanense*	FCATAS2708T	China	OL467292	OL584227	Ma et al. (2022)
*Jackrogersella cohaerens*	CBS 119,126	Germany	KY610396	KY624314	Wendt et al. (2018)
*J. multiformis*	CBS 119,016 ET	Germany	KC477234	KX271262	Kuhnert et al. (2014)
*Pyrenopolyporus hunteri*	MUCL 52,673 ET	Ivory Coast	KY610421	KU159530	Wendt et al. (2018)
*Py.laminosus*	MUCL 53305T	Martinique	KC968934	KC977292	Wendt et al. (2018)
*Py. nicaraguensis*	CBS 117,739	Burkina Faso	AM749922	KC977272	Wendt et al. (2018)
*Rhopalostroma angolense*	CBS 126,414	Ivory Coas	KY610420	KX271277	Kuhnert et al. (2014)
*Thamnomyces dendroidea*	CBS 123,578 T	French Guiana	FN428831	KY624313	Stadler et al. (2014)
*X. arbuscula*	CBS 126,415	Germany	KY610394	KX271257	Wendt et al. (2018)

Phylogenetic analysis was conducted using a combined dataset of ITS and *β-tubulin* sequences. A phylogenetic tree was constructed using both the maximum likelihood (ML) and Bayesian inference (BI) methods. An ML analysis was performed with 25 rate categories and 1000 bootstrap (BS) replicates under the GTRCAT model of nucleotide substitution, using RAxML-HPC2 version 8.2.12 (Felsenstein, 1985, Stamatakis, 2006) via the CIPRES web portal. For the BI analysis, the best-fit substitution models were determined using the Akaike information criterion (AIC) in jModelTest version 2.1.10 (Darriba et al., 2012): GTR for ITS and *β-Tubulin*. BI was carried out using MrBayes version 3.2.6 (Ronquist et al., 2012), with six simultaneous Markov chains run for one million generations, starting from random trees and sampling every 1000 generations. The first 20 % of trees, representing the burn-in phase, were discarded. The remaining trees were used to calculate posterior probabilities (PPs) in a majority rule consensus tree. The resulting phylogenetic trees were visualized using FigTree version 1.4.0 (Rambaut, 2012).

### 2.5 Extraction and purification of bioactive compounds from fungal endophytes

The fungal endophytes were cultured on potato dextrose agar (PDA) at 30 °C for 7 days. Next, 0.5 cm mycelial plugs (using a No. 5 cork borer) from 250 plugs were inoculated into 5000 mL Erlenmeyer flasks containing 2500 mL of potato dextrose broth (PDB) and incubated at room temperature for 30 days under static conditions. After 30 days, the fungal culture was filtered to remove the mycelium. The mycelium was then dried in a hot air oven at 55 °C for 5 days. The filtered broth and dried mycelium were subsequently extracted twice with an equal volume of ethyl acetate. The organic phases were evaporated to dryness at 40 °C using a rotary vacuum evaporator under reduced pressure. The fungal crude extract was then weighed. The crude extract of each endophytic fungal isolate was subsequently dissolved in dimethyl sulfoxide (DMSO) and then diluted with sterile distilled water to a concentration of 20 mg/mL. The effects of the fungal crude extracts on the growth of R. solanacearum were tested using the disc diffusion method. The crude ethyl acetate extract of the endophytic fungi from Compound 1 was subjected to repeated separations using column chromatography with an appropriate stationary phase and solvent system, as previously determined using thin layer chromatography (TLC). The crude extracts were then purified using silica gel column chromatography and eluted with a gradient of ethyl acetate and n-hexane or an isocratic system to obtain fractions using TLC analysis (Suebrasri et al., 2020).

### 2.6 Structure elucidation of the bioactive compound

Nuclear magnetic resonance (NMR) and mass spectrometry were used to predict the chemical structure of the isolated antifungal compounds. The compounds were analyzed using ¹H and ¹³C NMR spectra obtained on a Varian Mercury Plus 400 spectrometer. Chemical shifts are reported in δ (ppm), with CDCl₃ or CD₃OD as the solvent and residual CHCl₃ as the internal standard. The ¹H and ¹³C NMR data were recorded, detailing the number of protons, multiplicity, and coupling constants (J in Hz) (Suebrasri et al., 2020).

### 2.7 SEM analysis of the effects of the compounds on bacterial cells

A scanning electron microscope (SEM) was used to detect changes on the bacterial cell surface. Therefore, the effects of the compounds on the cell structure of the bacterial pathogen *R. solanacearum* were observed via SEM. To prepare the sample, the bacteria grown in nutrient broth for 18 h were centrifuged at 12,000 rpm and 4 °C for 2 min. The bacterial cells were washed with phosphate-buffered saline solution twice. The bacterial cell turbidity at 0.08–0.10 (10^6^ CFU/mL) was subsequently adjusted using a spectrophotometer at 600 nm. The 50 μL of the bacterial mixture was transferred to a microcentrifuge tube with 50 μL of the compound at 1 μg/mL, which was dissolved in 10 % DMSO. The mixture was incubated at 37 °C for 1 hour. After incubation, the solution was dropped on a nuclear pore polycarbonate membrane and fixed with 2.5 % glutaraldehyde overnight at 4 °C. Subsequently, the samples were dehydrated through a series of ethanol dilutions (50, 60, 70, 80, 90, 95, and 100 %) for 10 min at each gradient. The samples were placed onto copper stubs and coated with gold prior to observation using a scanning electron microscope (SEM) (LEO, Electron Microscopy Ltd., Cambridge, UK).

### 2.8 Disease suppression assay in tomato

The pot experiment was conducted in a greenhouse at the Department of Microbiology, Faculty of Science, Khon Kaen University, Khon Kaen, Thailand. The sandy loam soil used in the experiment had the following chemical properties: pH 7.26, electrical conductivity (EC) of 0.043 dS/m, 6.4 g kg⁻¹ of soil organic matter, 240 mg kg⁻¹ of total nitrogen (N) (with a soil C/N ratio of approximately 13), 146 mg kg⁻¹ total of phosphorus (P), 428 mg kg⁻¹ of extractable potassium (K), 61 mg kg⁻¹ of available phosphorus, 50 mg kg⁻¹ of exchangeable potassium, 655 mg kg⁻¹ of extractable calcium (Ca), and 50 mg kg⁻¹ of extractable sodium (Na). The soil was sterilized twice through autoclaving at 121 °C for 120 min at a pressure of 15 psi and kept at room temperature until it was used in the pot experiment. The pot design was conducted using a completely randomized design (CRD) with five replications as follows: T1, uninoculated with the pathogen; T2, pathogenic *R. solanacearum*; T3, crude extract of the endophytic fungus *H. saxatilis* KKU-KHP 01 + *R. solanacearum*; T4, endophytic fungus *H. saxatilis* KKU-KHP 01 + *R. solanacearum*; and T5, Bactericide.

The tomato seedlings were grown for 30 days and then transferred to 12-inch diameter pots filled with sterilized soil. Three days after planting, 50 mL of Hoagland’s solution was added. Fourteen days after planting, a bacterial culture of *R. solanacearum* at 0.5 McFarland, with a volume of 10 mL per pot, was prepared. The tomato roots were subsequently cut with a scalpel and inoculated with 10 mL of the bacterial culture. After 3 days of *R. solanacearum* infection, the endophytic fungus *H. saxatilis* KKU-KHP 01 from sorghum seeds was prepared, and the plants were treated with 10 mL of the crude extract of the endophytic fungus *H. saxatilis* KKU-KHP 01 at a concentration of 32 μg/mL. After seven days of *R. solanacearum* inoculation, 10 mL of the endophytic fungus *H. saxatilis* KKU-KHP 01 crude extract at a concentration of 32 μg/mL was added again. Regular watering was resumed (Kemboi et al., 2022).

### 2.9 Data collection

The plant data were collected 14 days after the inoculation of *R. solanacearum* to calculate the disease severity index (DSI) and disease reduction (DR) percentages of each treatment (Chiang et al., 2017). The severity of bacterial wilt disease was assessed using the a rating scale (0–5) from Kemboi et al. (2022), where 0 = no symptoms; 1 = 1–25 % of the leaves wilted; 2 = 26–50 % of the leaves wilted; 3 = 51–75 % of the leaves wilted; 4 = more than 75 % but less than 100 % of the leaves wilted; and 5 = all the leaves wilted and the tomato died (Kemboi et al., 2022).

### 2.10 Statistical analysis

Analysis of variance (ANOVA) was performed using SPSS version 26 (IBM). The data were analyzed using the least significant difference (LSD) test at a 0.05 probability level to determine significant differences between the treatments.

## 3 Results

### 3.1 Isolation and screening of the effects of fungal endophytes on bacterial growth

**Fig. 1:**
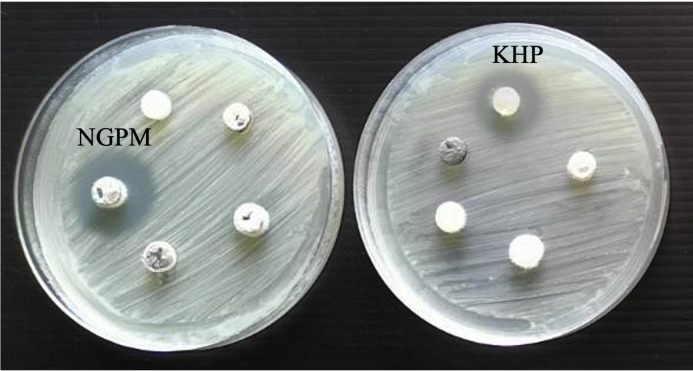
The antagonistic activity of the endophytic fungi against *R. solanacearum* was determined using an agar plug diffusion test.

The 48 endophytic fungal isolates were isolated from 20 medicinal plants collected from a mountain in the Lao Hai Ngam subdistrict, Kuchinarai District, Kalasin Province, Thailand (Supplementary Table S1). All fungal endophytes were assayed for their antibacterial activity against *R. solanacearum* using the agar plug diffusion technique. Potential biological control activity of the fungal endophytes was observed against *R. solanacearum* using the agar plug diffusion test. The results indicate that among several endophytic fungi, the fungal endophyte isolates NGPM and KHP, which were isolated from *Acanthus ebracteatus* and *Gardenia saxatilis* (Geddes), respectively, could produce a clear zone around the agar plug at 18 mm and 15 mm, as shown in Fig. 1. Consequently, both endophytic fungal isolates were further identified.

### 3.2 Identification of fungal endophytes

The endophytic fungus KHP, cultured on oatmeal agar, exhibited slow growth at 28 °C for 14 days. The colony appeared white with fluffy mycelia. In contrast, when grown on potato dextrose agar under the same conditions, the colonies displayed yellowish‒white coloration. Microscopic examination revealed hyphal extremities and chlamydospores. The endophytic fungus was subsequently identified based on the ITS and *β-Tubulin* sequences. The results of the BLAST search for sequence similarity of the ITS and *β-Tubulin* regions are presented in Table 2. A comparison of the ITS and TUB2 sequences between the endophytic fungus KHP and the reference strain revealed that the sequence similarity value was less than 93.02 %. Based on these results, the endophytic fungal strain KHP can likely be classified as a new species. Therefore, phylogenetic placement was observed using multigene phylogenetic analyses.

**Table 2:** The similarity of the ITS and *β-Tubulin* sequences between the fungal strains in this study and the top five related species of *Hypoxylon* in the GenBank database.

Related species of *Hypoxylon*	Sequence similarity value ( %)	
	ITS	*TUB2*
*H. begae* YMJ 215	93.02	93.00
*H. aveirense* CMG 29	88.80	87.03
*H. griseobrunneum* CBS:331.73	87.13	86.40
*H. anthochroum* YMJ 9	87.00	86.84
*H. diperithecium* FCATAS4226	86.80	87.77

**Fig. 2:**
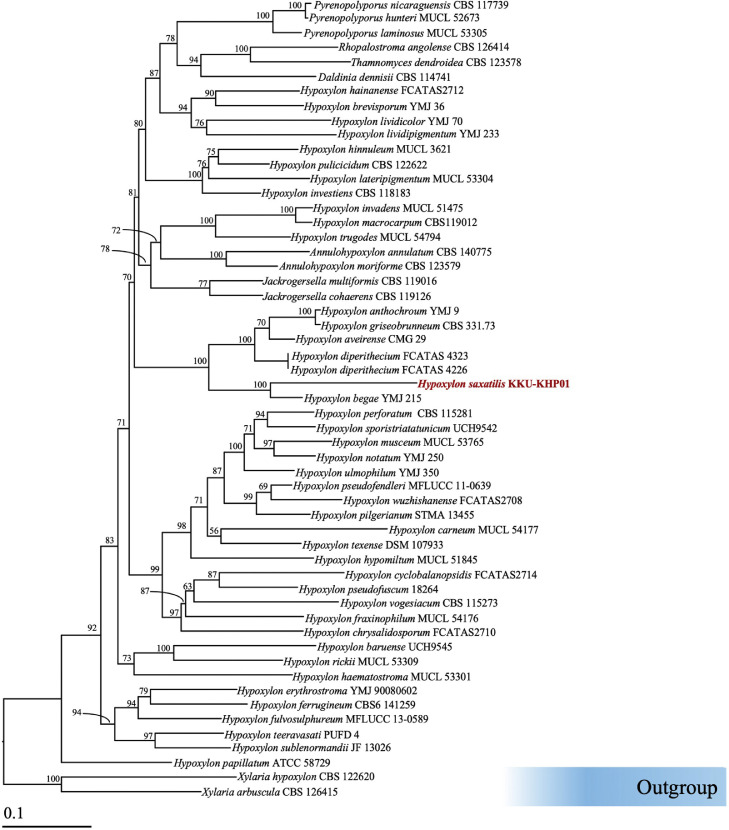
Phylogenetic tree derived from maximum likelihood analysis of 55 fungal strains harboring combined ITS and *β-Tubulin* genes. *Xylaria hypoxylon* CBS 122,620 and *X. arbuscula* CBS 126,415 were used as the outgroup. The numbers above the branches represent the maximum likelihood bootstrap percentages. Bootstrap values ≥70 %. The scale bar represents the expected number of nucleotide substitutions per site. The sequences of the fungal strains obtained in this study are in red.

The phylogenetic tree generated from the maximum likelihood (ML) analysis is shown in Fig. 2. This tree placed the endophytic fungal strain KHP in a distinct clade, separated from previously known *Hypoxylon* species, with strong support (BS = 100 %, PP = 1.00). Based on these results, the endophytic fungal strain KHP was designated as H. saxatilis KKU-KHP01.

### 3.3 Biological activity of crude extracts from fungal endophytes

The ethyl acetate crude extracts of *Hypoxylon* sp. NGPM and *Hypoxylon saxatilis* KKU-KHP 01 were tested for antagonistic activities against *R. solanacearum,* a causative agent of bacterial wilt in tomatoes. The crude extract from both the mycelia and fermentation broth of fungal endophytes has antagonistic effects on *R. solanacearum.* However, fermentation broth effectively inhibited the growth of *R. solanacearum*, especially the fermentation broth of the endophytic fungus *H. saxatilis* KKU-KHP 01. Therefore, the crude extract of the endophytic fungus *H. saxatilis* KKU-KHP 01 was further analyzed to elucidate the structures of the bioactive compounds.

### 3.4 Structure elucidation of the bioactive compound

The EtOAc crude extract of *H. saxatilis* KKU-KHP 01 was subjected to purification using silica gel chromatography (CC) with gradients of EtOAc in *n*-hexane, resulting in four fractions, KHP1–KHP4. All the fractions were tested for their activity against *R. solanacearum.* Fraction KHP2 was purified by CC and eluted with CH_2_Cl_2_−hexanes (2:8, v/v), yielding compound **1** as a colorless oil.

**Fig. 3:**
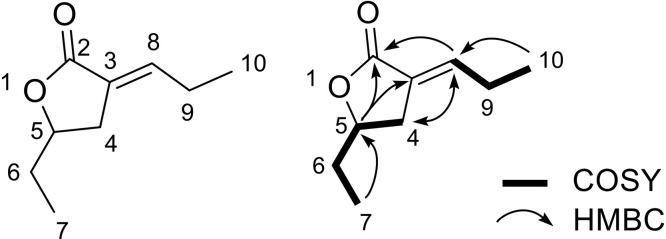
Key COSY and HMBC correlations of compound **1.**

Compound **1** was obtained as a colorless oil. Its molecular formula (C_9_H_14_O_2_) was deduced from the positive-ion HRESIMS *m/z* 155.1071 [*M* + *H*]^+^, (calcd for 155.1067, C_9_H_15_O_2_^+^). The ^1^H NMR spectrum (Table 3) revealed an olefinic proton resonance at δ_H_ 6.53 (m), an oxygenated methine resonance at δ_H_ 4.96 (m), diastereotopic methylene resonances at δ_H_ 2.84 (dd, 16.5, 7.5) and 2.33 (d, 16.5), two methylene resonances at δ_H_ 1.59 (m) and 2.06 (quintet, 7.5), and two methyl resonances at δ_H_ 0.95 (t, 7.5) and 0.86 (t, 7.5) ppm. The ^13^C NMR spectra (Table 3) revealed ten carbon resonances, including one ester carbonyl resonance at δ_C_ 170.9; two olefinic resonances at δ_C_ 141.5 and 125.8; one oxymethine resonance at δ_C_ 78.6; three methylene resonances at δ_C_ 30.4, 29.2, and 23.2; and two methyl resonances at δ_C_ 12.4 and 8.8 ppm. The planar structure of compound **1** was determined through analysis of its COSY, HSQC, and HMBC spectra. The key correlations are shown in Fig. 3. The COSY spectrum revealed two spin systems: H-4/H-5/H-6/H-7 and H-8/H-9/H-10.

**Table 3:** ^1^H (400 MHz) and ^13^C NMR (100 MHz) Spectroscopic Data of **1.**

No.	**1** [mult, *J* in Hz] in CDCl_3_	
	*δ*_H_	*δ_C_*
1	–	–
2	–	170.9
3	–	125.8
4	2.84 (dd, 16.5, 7.5) 2.33 (d, 16.5)	30.4
5	4.93 (m)	78.6
6	1.59 (m)	29.2
7	0.86 (t, 7.5)	8.8
8	6.53 (m)	141.5
9	2.06 (quintet, 7.5)	23.2
10	0.95 (t, 7.5)	12.4

The HMBC spectrum of H-5 to C-2 and C-3, H-8 to C-2 and C-4, and H-10 to C-8 demonstrated that the chain was located at C-3. Moreover, HMBC correlations were observed between H-7 and C-5, H-5 and C-2, and C-3, indicating that this chain was connected to C-5. Therefore, the core structure of compound **1** was identified as tetrahydrofuran. Moreover, compound **1** was therefore identified as an undescribed secondary metabolite from natural sources.

### 3.5 SEM studies of the effects of the compounds on bacterial cells

**Fig. 4:**
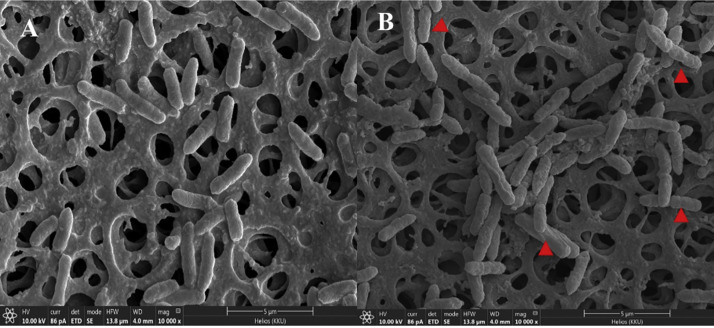
Effects of compound **1** on the structural changes of *R. solanacearum* cells using SEM: (A) *R. solanacearum* cells without exposure to compound **1**, (B) *R. solanacearum* cells exposed to compound **1**, (arrowhead) bacterial cells showing wrinkles after exposure to compound **1**.

SEM revealed the effects of compound **1** from *H.* saxatilis KKU-KHP 01 on the cell structure of *R. solanacearum. C*ompound **1** caused changes to the bacterial cell surface, with the areas exposed to the compound appearing wrinkled. Therefore, the inhibition of the growth of the pathogenic bacterium *R. solanacearum* may be due to the ability of pure compound **1** to damage the bacterial cell wall, as shown in Fig. 4.

### 3.6 Disease suppression assay

**Fig. 5:**
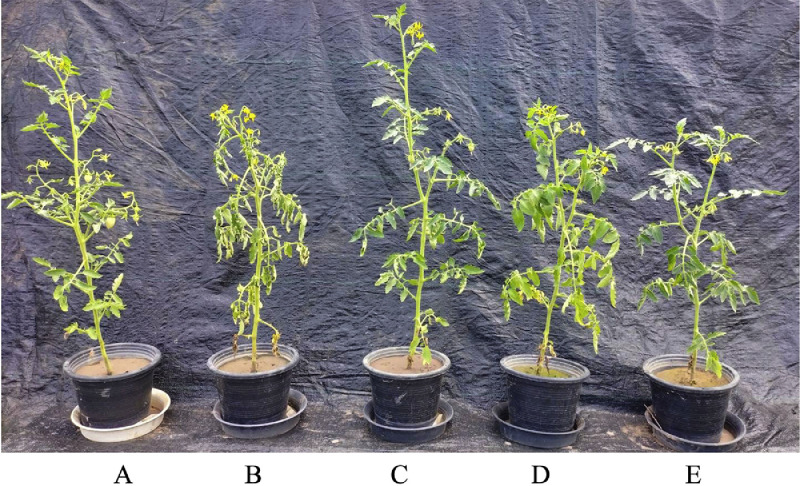
Effects of the crude extract and the endophytic fungus *H. saxatilis* KKU-KHP 01 on the inhibition of wilt disease in tomatoes caused by *R. solanacearum* under greenhouse conditions 14 days after inoculation with *R. solanacearum*. (A) Negative control, uninoculated with the pathogen; (B) positive control, inoculated with the pathogen *R. solanacearum*; (C) plants inoculated with *R. solanacearum* and treated with the crude extract of the endophytic fungus *H. saxatilis* KKU-KHP 01; (D) plants inoculated with *R. solanacearum* and co-inoculated with the endophytic fungus *H. saxatilis* KKU-KHP 01; and (E) bactericide.

Fourteen days after *R. solanacearum* infection, the percentage of disease severity index and disease reduction were calculated. The results reveal that when *R. solanacearum* was inoculated and treated with the crude extract of the endophytic fungus *H. saxatilis* KKU-KHP 01 at a concentration of 32 μg/mL, the disease severity index (DSI) was 16 %. This value was significantly lower than that of the co-inoculation of the endophytic fungi *H. saxatilis* KKU-KHP 01 and *R. solanacearum*, which had a DSI of 36 % (*p* < 0.05). Additionally, the crude extract of the endophytic fungus *H. saxatilis* KKU-KHP 01 was able to reduce the severity of wilt disease caused by *R. solanacearum* by 83.33 %, which was significantly higher (*p* < 0.05) than that resulting from the co-inoculation of the endophytic fungus and *R. solanacearum* and bactericide, resulting in disease reduction percentages of 62.50 % and 70.00 %, respectively, as shown in Table 4 and Fig. 5.

**Table 4:** Effect of the crude extract and the endophytic fungus *H. saxatilis* KKU-KHP 01 on the inhibition of wilt disease in tomatoes caused by *R. solanacearum* under greenhouse conditions 14 days after inoculation with *R. solanacearum*.

Treatment	Disease severity index ( %)	Disease reduction ( %)
Uninoculation with the pathogen	0.00± 0.00	0.00± 0.00
*R. solanacearum*	96.00 ± 0.08^a^	0.00 ± 0.00^c^
Crude extract of *H. saxatilis* KKU-KHP 01 + *R. solanacearum*	16.00 ± 0.08^c^	83.33 ± 0.09^a^
Endophytic fungus *H. saxatilis* KKU-KHP 01+*R. solanacearum*	36.00± 0.16^b^	62.50 ± 0.17^b^
Bactericide	30.00±0.12^b^	70.00±0.13^b^

The disease severity index (DSI) and disease reduction (DR) were calculated 14 days after the inoculation of *R. solanacearum*, and the values are the means (±SD) of the percentage of *R. solanacearum* inhibited. The results represent the average number of tomatoes planted in the pots, with five replications per treatment. Different letters indicate statistically significant differences between the data according to the LSD test (*p* < 0.05).

## 4 Discussion

The ability of endophytic fungi to inhibit bacterial wilt disease caused by *R. solanacearum* in tomatoes was tested. Twenty species of medicinal plants were collected from mountains in the Lao Hai Ngam subdistrict, Kuchinarai District, Kalasin Province, Thailand. A total of 48 endophytic fungal isolates were obtained. This finding is consistent with recent research highlighting that medicinal plants serve as reservoirs for diverse endophytic fungi with potential bioactive properties (Gupta et al., 2023). The inhibitory effect of these fungal isolates against *R. solanacearum* supports their promising role as biological control agents in managing bacterial wilt disease in tomatoes. Recent studies have emphasized the ability of endophytic fungi to produce antimicrobial compounds and induce systemic resistance in host plants, thereby suppressing pathogenic bacteria (Lee et al., 2022; Zhang et al., 2024). Endophytic fungi are microorganisms that live within plant tissues without causing disease or harm to the host plant (Smith and Doe, 2023). These fungi can protect plants from pathogen invasion, such as *Sclerotium rolfsii*, which causes root rot disease (Suebrasri et al., 2020). Endophytic fungi have also been used to inhibit Fusarium wilt disease in tomatoes caused by *Fusarium oxysporum* f. sp. *lycopersici* (Muhorakeye et al., 2024) and to suppress bacterial spot disease in tomatoes caused by *Xanthomonas vesicatoria* (Rashid, 2021). However, research on the role of endophytic fungi in the inhibition of *R. solanacearum* is relatively limited. Therefore, the antagonistic activity of 48 endophytic fungal isolates against *R. solanacearum* was evaluated using the agar plug diffusion test. Two fungal isolates, NGPM and KHP, inhibited *R. solanacearum*. These endophytic fungi were isolated from the midribs of Acanthus ebracteatus (NGPM) and the petioles of *Gardenia saxatilis* (Geddes) (KHP). The isolates were subsequently classified based on their morphological and molecular characteristics. The fungi were identified as *Hypoxylon* sp. NGPM and *Hypoxylon* sp. KHP, with 100 % and 95.52 % similarity, respectively, based on ITS1–4 primers. However, based on the similarity of the TUB2 sequence to that of the reference strains, including *H. begae, H. aveirense, H. griseobrunneum, H. anthochroum*, and *H. diperithecium*, the similarity did not meet the TUB2 threshold of ≥ 87.77 % (Table 2). Therefore, *H. saxatilis* can be considered a new and different species from these five species. Moreover, the secondary metabolites from these two endophytic fungi were extracted using both fermentation broth and mycelia. Notably, the fermentation broth had a greater inhibitory effect on *R. solanacearum* than did the mycelial extracts. Between the two fungi, *H. saxatilis* KKU-KHP 01 exhibited more efficient inhibition. This difference can be attributed to the fact that the fermentation broth contains extracellular metabolites secreted by the fungi during growth, which are often more bioavailable and potent against pathogens (Kusari et al., 2012). The secondary metabolites were purified using column chromatography and identified as compound **1**, a tetrahydrofuran (THF) derivative. NMR analysis confirmed that compound **1** was the first report of a new naturally occurring secondary metabolite. In addition, the minimum inhibitory concentration (MIC) and minimum bactericidal concentration (MBC) of compound **1** were found to be 0.5 µg/mL and 1.0 µg/mL, respectively, which were lower than those of the two antibiotics used as positive controls, chloramphenicol and kanamycin. SEM analysis revealed that *R. solanacearum* exposed to tetrahydrofuran (THF) exhibited cell wall wrinkles, a well-known characteristic of THF toxicity against bacteria (Yao et al., 2010). The general mechanism of action of THF includes damage to the bacterial cell membrane (Zhang et al., 2014), protein denaturation (Hochkoeppler et al., 2007), inhibition of bacterial metabolism by targeting enzymes involved in energy production and growth (Tian et al., 2019), and DNA damage (Hedrick et al., 2014). Therefore, tetrahydrofuran (THF), which is secreted by the endophytic fungus *H. saxatilis* KKU-KHP 01, can potentially destroy the bacterial pathogen *R. solanacearum*. Moreover, this study is the first report on the use of the novel secondary metabolite tetrahydrofuran produced by the endophytic fungus *H. saxatilis* KKU-KHP 01 for inhibiting the growth of *R. solanacearum*. Previously reported, crude extracts from the endophytic fungus *Trichoderma* spp. reduced bacterial wilt severity in tomatoes caused by *R. solanacearum* by 60–75 % (Siddiqui et al., 2017), and crude extracts from the endophytic fungus *Penicillium* spp. reduced bacterial wilt severity by 40–60 % (Sarma et al., 2015). In the present study, the crude extract of *H. saxatilis* KKU-KHP 01 was evaluated under greenhouse conditions for its ability to reduce the severity of bacterial wilt disease caused by *R. solanacearum*. The results showed an 83.33 % reduction in disease severity, which was significantly higher than the treatment with bactericide and those in previous reports. Therefore, tetrahydrofuran isolated from the endophytic fungus *H. saxatilis* KKU-KHP 01 exhibits promising potential for controlling bacterial wilt disease in tomatoes under field conditions. Furthermore, this compound could be further developed into a commercial biocontrol agent for sustainable agricultural applications.

## 5 Conclusion

This study demonstrated that endophytic fungi isolated from medicinal plants, particularly *Hypoxylon saxatilis* KKU-KHP 01, possess strong antibacterial activity against *R. solanacearum*, the causative agent of wilt disease in tomatoes. Notably, *H. saxatilis* KKU-KHP 01 was identified as a novel fungal species capable of producing tetrahydrofuran, a compound not previously reported as a natural fungal metabolite. Tetrahydrofuran significantly inhibited the pathogen by disrupting bacterial cell wall integrity, as observed through scanning electron microscopy. Under greenhouse conditions, the crude extract of *H. saxatilis* KKU-KHP 01 reduced wilt severity by up to 83.33 %. These findings highlight the potential application of tetrahydrofuran as a biocontrol agent for managing bacterial wilt in tomato cultivation.

## Credit author statement

**Table:** 

Name	Credit
Thanapat Suebrasri	Conceptualization, Methodology, Investigation, Data analysis and Writing - Original Draft
Wasan Seemakram	Conceptualization, Methodology, Investigation, data analysis and funding acquisition
Awat Wisetsai	Investigation some experiment and data analysis
Thanawan Gateta	Investigation some experiment and data analysis
Sutarin Preepram	Investigation some experiment and data analysis
Phornnapa Saentao	Investigation some experiment and data analysis
Sophon Boonlue	Conceptualization, Writing- Reviewing and Editing, funding acquisition

## Funding

This research was funded by a grant from the Fundamental Fund of 10.13039/501100004071Khon Kaen University through the National Science, Research and Innovation Fund (NSRF). We are grateful to the Plant Genetic Conversion Project under the Royal Initiative of Her Royal Highness Princess Maha Chakri Sirindhorn, Khon Kaen University.

## Data availability statement

The datasets obtained and analyzed in the current study are available from the corresponding author upon reasonable request.

## Declaration of competing interest

The authors declare that they have no known competing financial interests or personal relationships that could have appeared to influence the work reported in this paper.

## Data Availability

No data was used for the research described in the article.

## Appendix Supplementary materials

Image, application 1
