# Supplementary material for: Novel secondary metabolite from a new species of Hypoxylon saxatilis sp. nov. for suppressing bacterial wilt in tomato
Source: Curr Res Microb Sci. 2025 Jul 20;9:100445. doi: 10.1016/j.crmicr.2025.100445 (PMC12312046; doi:10.1016/j.crmicr.2025.100445)
Supplement: Supplementary file 1 [file mmc1.docx]

**Table S1**. The host plant and the number of endophytic fungi isolated from the medicinal plants.

| **Host plant** | **Code** | **samples** | **Total fungal isolates** |  |
| --- | --- | --- | --- | --- |
| *Phyllanthus niruri* | LB | 10 | 2 | |
| *Aganosma marginata* (Roxb.) | ST | 10 | 9 | |
| *Vernonia cinerea* Less. | MP | 10 | 5 | |
| ***Acanthus ebracteatus*** | NGPM | 10 | 1 | |
| *Tiliacora triandra* | YN | 10 | 4 | |
| *Drynaria quercifolia* | KT | 10 | 1 | |
| *Bauhinia scandens* L. | KD | 10 | 2 | |
| *Memecylon edule* Roxb. | MA | 10 | 3 | |
| *Sindora siamensis* | MT | 10 | 1 | |
| ***Gardenia saxatilis* (Geddes)** | KH | 10 | 2 | |
| *Aspidistra sutepensis* | NL | 10 | 2 | |
| *Cissampelos pareira* | KN | 10 | 1 | |
| *Polyalthia debilis* | TK | 10 | 3 | |
| *Gardenia thailandica* | PP | 10 | 2 | |
| *Smilax perfoliata* | PK | 10 | 2 | |
| *Acanthus ebracteatus* | NG | 10 | 1 | |
| *Bauhinia acuminata* | KW | 10 | 1 | |
| Sphaerocoryne lefevrei | LD | 10 | 2 | |
| *Pouzolzia pentandra* | KOB | 10 | 3 | |
| *Barleria cristata* L. | ANG | 10 | 1 | |
| **Total** | **20** | **200** | **48** | |
